# Glyceraldehyde 3-Phosphate Dehydrogenase-Telomere Association Correlates with Redox Status in *Trypanosoma cruzi*

**DOI:** 10.1371/journal.pone.0120896

**Published:** 2015-03-16

**Authors:** Ricardo Pariona-Llanos, Raphael Souza Pavani, Marcelo Reis, Vincent Noël, Ariel Mariano Silber, Hugo Aguirre Armelin, Maria Isabel Nogueira Cano, Maria Carolina Elias

**Affiliations:** 1 Laboratório Especial de Ciclo Celular, Instituto Butantan, São Paulo, Brazil; 2 Center of Toxins, Immune Response and Cell Signaling—CeTICS, Instituto Butantan, São Paulo, Brazil; 3 Unit for Drug Discovery—Departamento de Parasitologia, Instituto de Ciências Biomédicas, Universidade de São Paulo, São Paulo, Brazil; 4 Departamento de Bioquímica, Instituto de Química, Universidade de São Paulo, São Paulo, Brazil; 5 Departamento de Genética, Instituto de Biociências, Universidade Estadual Paulista Júlio de Mesquita Filho—UNESP, Botucatu, Brazil; Federal University of São Paulo, BRAZIL

## Abstract

Glyceraldehyde 3-phosphate dehydrogenase (GAPDH) is a classical metabolic enzyme involved in energy production and plays a role in additional nuclear functions, including transcriptional control, recognition of misincorporated nucleotides in DNA and maintenance of telomere structure. Here, we show that the recombinant protein *T*. *cruzi* GAPDH (rTcGAPDH) binds single-stranded telomeric DNA. We demonstrate that the binding of GAPDH to telomeric DNA correlates with the balance between oxidized and reduced forms of nicotinamide adenine dinucleotides (NAD^+^/NADH). We observed that GAPDH-telomere association and NAD^+^/NADH balance changed throughout the *T*. *cruzi* life cycle. For example, in replicative epimastigote forms of *T*. *cruzi*, which show similar intracellular concentrations of NAD^+^ and NADH, GAPDH binds to telomeric DNA *in vivo* and this binding activity is inhibited by exogenous NAD^+^. In contrast, in the *T*. *cruzi* non-proliferative trypomastigote forms, which show higher NAD^+^ concentration, GAPDH was absent from telomeres. In addition, NAD^+^ abolishes physical interaction between recombinant GAPDH and synthetic telomere oligonucleotide in a cell free system, mimicking exogenous NAD^+^ that reduces GAPDH-telomere interaction *in vivo*. We propose that the balance in the NAD^+^/NADH ratio during *T*. *cruzi* life cycle homeostatically regulates GAPDH telomere association, suggesting that in trypanosomes redox status locally modulates GAPDH association with telomeric DNA.

## Introduction

Glyceraldehyde 3-phosphate dehydrogenase (GAPDH) is a classical metabolic enzyme involved in energy production but is also thought of as a “moonlighting protein”, an idea first proposed by Jeffery [1]. This class of proteins includes multitasking proteins that follow the paradigm of one protein performing multiple functions. In mammalian cells, GAPDH displays distinct membrane, cytosolic and nuclear localization patterns [2] and the DNA binding protein of GAPDH was described around 30 years ago [3]. With respect to its nuclear function, it has been shown that a nuclear form of GAPDH (p38/glyceraldehyde-3-phosphate dehydrogenase) is a key component of the OCA-S complex, which is a transcription cofactor complex that directly stimulates the transcription of the human H2B gene during S-phase [4]. In addition, it has also been shown that intracellular NAD^+^/NADH ratios determine the rate of H2B transcription [5], since, p38/GAPDH (OCA-S) disengaged from the H2B promoter upon redox perturbation [6].

Nuclear GAPDH is also involved in the regulation of telomere structure in mammalian cells. Partial inhibition of GAPDH expression in mammalian cells by small interference RNA resulted in the rapid shortening of telomeres [7], whereas the overexpression of nuclear GAPDH resulted in the protection of telomeric DNA in response to anti-cancer drugs [8]. Mammalian GAPDH is thought to bind telomeric DNA via its NAD^+^ binding site because mutations in this site almost completely abolish GAPDH-telomere interactions [8].

*Trypanosoma cruzi* is a pathogenic protozoan that is the causative agent of Chagas’ disease, whose infection rates estimated at between 8–11 million people [9]. This protozoan parasite appeared early during the evolution of eukaryotes and exhibits peculiar characteristics such as gene transcription by polycistronic units that are processed by a trans-splicing reaction [10–12]. Therefore, we first examined whether GAPDH is a multitasking protein in these early divergent eukaryotes and tested its ability to associate with telomeric DNA. In addition, because this parasite is exposed to environmental changes during its life cycle and exhibits flexible metabolic activity, we tested GAPDH-telomeric DNA associations in parasite cells maintained in different cellular redox state. We show that GAPDH-telomere interaction occurs in trypanosomes and that their redox state is related to the level of GAPDH-telomere association in *T*. *cruzi*. We demonstrate that the NAD^+^/NADH balance and the ability of GAPDH to associate with telomeric DNA changes during the *T*. *cruzi* life cycle. Moreover, NAD^+^ abolishes physical interaction between recombinant-GAPDH and synthetic telomere-oligonucleotide in a cell-free system, mimicking exogenous NAD^+^ that reduces GAPDH-telomere interaction *in vivo*. Taken together, these data suggest that the NAD^+^/NADH balance may control the association between GAPDH and telomeric DNA in trypanosomes so that telomere maintenance and metabolic control are coupled and occurred early during evolution.

## Materials and Methods

### Cell culture

*T*. *cruzi* Y strain was used in this study. Epimastigote forms were maintained in liver infusion tryptose medium containing 10% fetal bovine serum at 28°C [13]. Trypomastigotes were obtained from infected LLC-MK2 cells (American Type Culture Collection, Rockville, MD) grown in Dulbecco’s modified Eagle’s medium containing 10% fetal bovine serum at 37°C and 5% CO_2_. Trypomastigotes were collected from the extracellular medium 5–7 days after infection and were recovered after centrifugation at 2,000 × g for 10 min.

### Determination of intracellular NAD^+^ and NADH levels

Cells were mock treated or treated for 10 min with 500 μM NAD^+^ or NADH. The NAD^+^ and NADH intracellular concentrations were measured using an NAD^+^/NADH quantification kit (Bio Vision).

### Fractionation of cellular proteins

To fractionate cellular proteins, 10^8^ *T*. *cruzi* epimastigotes or trypomastigotes were treated with 100 μl of extraction buffer [(0.1% Triton X-100, 10 mM Tris-HCl pH 7.4, 100 mM NaCl, 300 mM sucrose, 3 mM MgCl_2_, 50 mM NaF, 1 mM Na_3_VO_4_, 0.5 mM PMSF, and EDTA-free Complete protease inhibitor cocktail (Roche)] for 10 min at 4°C with agitation. Samples were pelleted, and supernatants were saved and labeled as soluble fraction 1. Cells were treated with the extraction buffer again and the second set of supernatants was saved (soluble fraction 2). Pellets were then treated with DNase I (5 units per 10^6^ cells) (Fermentas) for 30 min at room temperature. Samples were subsequently pelleted and the supernatants were saved and designated as the DNase I released fraction (DNA binding protein 1). Cells were treated with DNase I again and the second round of supernatant was saved (DNA binding protein 2). Samples were resolved by SDS-PAGE and analyzed by Western blot.

### Western blot

Immunoblotting was performed using sodium dodecyl sulfate (SDS) extracts containing 1 x 10^7^ cells per lane. Membranes were incubated with mouse anti-GAPDH [14] diluted 1:1,000, mouse anti-histone H4 [15] diluted 1:1,000, or mouse anti-hsp70 [16] diluted 1:10,000. Secondary antibodies (anti-mouse) were diluted 1:2,000. Detection was performed by ECL (Amersham Biosciences) using standard protocols.

### Gel shift assay

Single (ss) and double stranded (ds) oligonucleotides (sequences are described in the figure legends) were labeled with DIG-11-ddUTP by terminal transferase using a Dig Gel Shift kit (Roche). To obtain ds oligonucleotides, both strands containing *Bam*HI restriction sites at both ends (to avoid mishybridization of the ds) were mixed at a molar ratio of 1:1 in the presence of TEN Buffer pH 8.0 (10 mM Tris-HCl, 1 mM EDTA, 1 mM NaCl). After an initial incubation at 95°C for 10 minutes, samples were maintained at room temperature. The gel shift assay was performed with the Dig Gel Shift kit (Roche) using 1 μg of rGAPDH (generously provided by Dr. Otavio Thiemann, Instituto de Física de São Carlos, USP) (purification and activity measurement of *T*. *cruzi* rGAPDH has been described [17,18]), 0.155 pmol of labeled oligonucleotide, 0.1 μg of poly-lysine, 1 μg poly[d(I-C)], 4 μl of 5X binding buffer (100 mM HEPES pH 6.0, 5 mM EDTA, 50 mM (NH_4_)_2_SO_4_, 5 mM DTT, Tween 20 1% (w/v), 150 mM KCl) and increasing concentrations (0.155, 1.55 and 15.5 pmol) of nonspecific or specific unlabeled DNA (see figure legends) in a final volume of 20 μl. Samples were maintained on ice for 15 min and then applied to a non-denaturing 6% gel (Acrylamide/Bis-Acrylamide,37.5:1) run at 80 V in 0.25X TBE buffer. Samples were then transferred onto a nylon membrane at 400 mA for 30 min in 0.25X TBE and were fixed by UV light for 15 min and detection of labeled oligonucleotides was performed using the Dig Gel Shift kit (Roche).

### Chromatin Immunoprecipitation

10^9^ cells obtained from an epimastigote growing culture (10^7^ cells/ml) or 10^9^ trypomastigotes obtained as described above, were collected, resuspended in 50 ml of PBS and 1% formaldehyde was added for 30 min at room temperature under agitation. Samples were then incubated for 5 minutes at room temperature in the presence of 125 mM glycine. Cells were subsequently washed with cold PBS containing 125 mM glycine and then washed twice in cold PBS. Cells were resuspended in 1 ml of lysis buffer [(50 mM HEPES pH 7.5, 140 mM NaCl, 1 mM EDTA, 0,1% sodium deoxycholate, 1% NP-40, 1 mM PMSF and Protease inhibitor cocktail tablets (Roche)] and sonicated for 8 pulses (10 sec each with an interval of 1 min) on ice to obtain DNA fragments of 500–700 bp. Samples were then centrifuged at 3,000 rpm for 40 min at 4°C and supernatants were utilized for immunoprecipitation reactions. Samples were incubated with mouse anti-GAPDH antibody [14] diluted 1:800, or with the same amount of pre-immune serum for 2 h at 4°C under agitation. Samples were centrifuged at 14,000 rpm for 15 min at 4°C and transferred to a new tube containing 40 μl of protein G sepharose beads (GE) previously incubated with 5 mg/ml BSA for 2 h at 4°C under agitation. Samples were then washed twice with 1 ml of wash buffer (10 mM Tris-HCl pH 8.0, 250 mM LiCl, 0,5% sodium deoxycholate, 1 mM EDTA and 0.5% NP-40) and twice with TE (10mM TriHCl pH 8.0, 1mM EDTA). Chromatin was eluted with 250 ml of elution buffer (50 mM Tris-HCl pH 8.0, 10 mM EDTA and 1% SDS) and incubated at 65°C overnight under agitation. Isolated DNA was then purified via phenol-chloroform extraction. DNA samples were heated to 90°C for 5 minutes and samples were loaded onto nylon membranes and cross-linked with UV. The G-rich telomeric DNA was detected using a labeled telomere probe [C-rich strand—(AATCCC)_6_] or a labeled non-specific probe of the same size (GCGGCCGCATGGACAGAGATTCACTTGTGG) by southern blotting using the *Gene Images Alkphos* kit (GE Healthcare) according to the manufacturer’s instructions.

### Imunofluorescence and imunofluorescence coupled FISH

Cells adhered on slides previously treated with polylysine were fixed with 4% paraformaldehyde for 25 min and then permeabilized with 0.1% Triton for 10 min. Cells were washed three times with PBS and incubated with anti-GAPDH diluted 1:800 in PBS-0.1% BSA for 1 h. After three washes with PBS, cells were incubated with anti-mouse antibody conjugated to Alexa 555 diluted 1:500 and 5μg/ml of DAPI in PBS-0.1% BSA for 45 min. In the case of FISH coupled assay, cells were then post-fixed with 4% cold formaldehyde for 20 min and washed three times with PBS. Cells were dehydrated with 70%, 80% and 90% ethanol and then air-dried. 10μl of hybridization solution (70% formamyde, 20 mM Tris-HCl pH 7.0 and 1% BSA) containing 1nmol of telomeric probe (5`TTAGGGTTA3’ FITC PNA) was added and slides were sealed using *Frame-Seal* (Bio-Rad). Slides were maintained at 95°C for 5 min and then incubated at 37°C overnight and then washed with Wash solution from Telomere PNA kit/FITC (Dako) for 5 min. Slides were again treated with 70%, 80% and 90% ethanol and air-dried. Slides were sealed in the presence of Vecta shield (Vector). Images were acquired through z-series of 0.2μm using lens of 100X 1.35NA using Cell R software in Olympus IX81 microscopy. Images were deconcolved using Autoquant X2.1.

### Computational methods

Quantification of the images was carried out using either Alliance (UVItec, Cambridge, UK) or ImageJ (Public Domain). The systems of differential equations that describe the kinetic models presented in this paper were implemented using MATLAB (MathWorks, Natick, MA), and the Sundials library for C [19]. The implemented codes are available upon request to the authors. Curve-fitting analyses were carried out employing parallel simulated annealing, using an implementation from [20].

## Results

### *T*. *cruzi* GAPDH binds telomeres *in vitro*

Trypanosomes are very early divergent eukaryotes; therefore, we can make inferences regarding the ancestral characteristics of eukaryotic cells by studying the biology of these parasites. Because GAPDH is a classical metabolic enzyme, we asked whether other recently described GAPDH functions could be detected in trypanosomes, such as the protection of chromosomal ends via its interactions with telomeric DNA.

**Fig 1:**
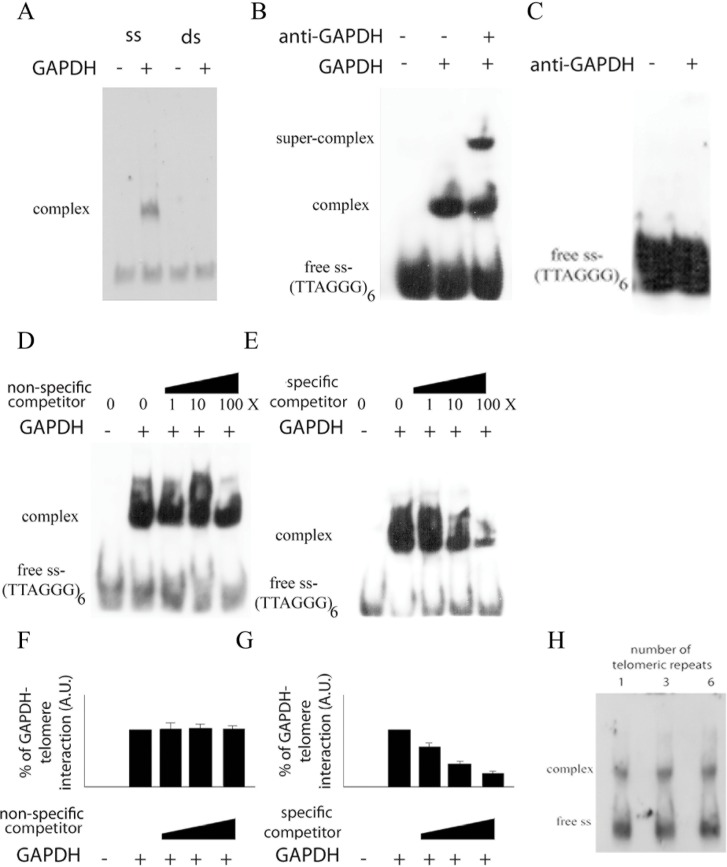
Trypanosome GAPDH interacts with telomeric DNA. (A) Oligos are labeled ss TTAGGG (ss) or ds TTAGGG (ds), both of which contain six tandem sequence repeat copies. They were incubated in the presence (+) or absence (-) of recombinant *T*. *cruzi* GAPDH. Samples were then analyzed by EMSA. (B) Labeled ss-TTAGGG containing six tandem sequence repeat copies were incubated with recombinant *T*. *cruzi* GAPDH only, or with both GAPDH and anti-GAPDH serum. The samples were analyzed by EMSA. (C) Labeled ss (TTAGGG)_6_ oligos were incubated in the presence (+) or absence (-) of an anti-GAPDH serum. The samples were subsequently analyzed by EMSA. (D) and (E) Labeled ss-TTAGGG containing 6 tandem sequence repeat copies were incubated with recombinant *T*. *cruzi* GAPDH in the presence of increasing concentrations (1, 10 and 100X, in relation to the labeled telomeric oligonucleotide) of an non-specific unlabeled competitor (GCGGCCGCATGGACAGAGATTCACTTGTGG) (D) or the presence of cold ss-(TTAGGG)_6_ (E). (F) and (G) The amount of complexes representing the telomere-GAPDH complex in D (F) or E (G) was estimated by ImageJ software. The graphs show the mean and standard deviation of three independent experiments. (H) Labeled ss-TTAGGG containing only one repeat unit or containing three or six tandem repeat sequences were incubated with 50 μM recombinant *T*. *cruzi* GAPDH and were analyzed by EMSA.

We tested whether the recombinant *T*. *cruzi* GAPDH enzyme (X52898) was able to bind to telomeres using an electrophoretic mobility shift assay (EMSA). In these assays, we used a ss oligonucleotide G-rich strand or a ds telomeric DNA both containing six TTAGGG telomeric repeats. rTcGAPDH was able to bind the ss (TTAGGG)_6_ oligonucleotide but was unable to bind the double stranded telomeric DNA (Fig. 1A). The results of the super shift assay corroborate the conclusion that the shift shown in Fig. 1A was due to the interaction between GAPDH and the ss telomere, since anti-GAPDH serum induced a supershift of the complex (Fig. 1B) and the anti-GAPDH antibody does not bind the ss telomeric oligonucleotide (Fig. 1C). Also, rTcGAPDH-telomeric/oligonucleotide interaction is not through his-tag fused to rTcGAPDH, because it was already demonstrated that his-tag does not associate with telomere sequence TTAGGG [21].

To test whether the rTcGAPDH-telomeric/oligonucleotide interaction was specific, we incubated rTcGAPDH with increasing concentrations of a non-labeled specific competitor (six tandem repeats of TTAGGG) or of a non-labeled nonspecific competitor (see figure legends). As shown in Fig. 1D-1G, the rTcGAPDH-telomeric/oligonucleotide interaction was disrupted only in the presence of the specific competitor, demonstrating the existence of a specific interaction between *T*. *cruzi* GAPDH and the telomeric G-rich strand. Moreover, we found that rTcGAPDH can form complexes with oligonucleotides containing one, three or six telomeric repeats (Fig. 1H).

**Fig 2:**
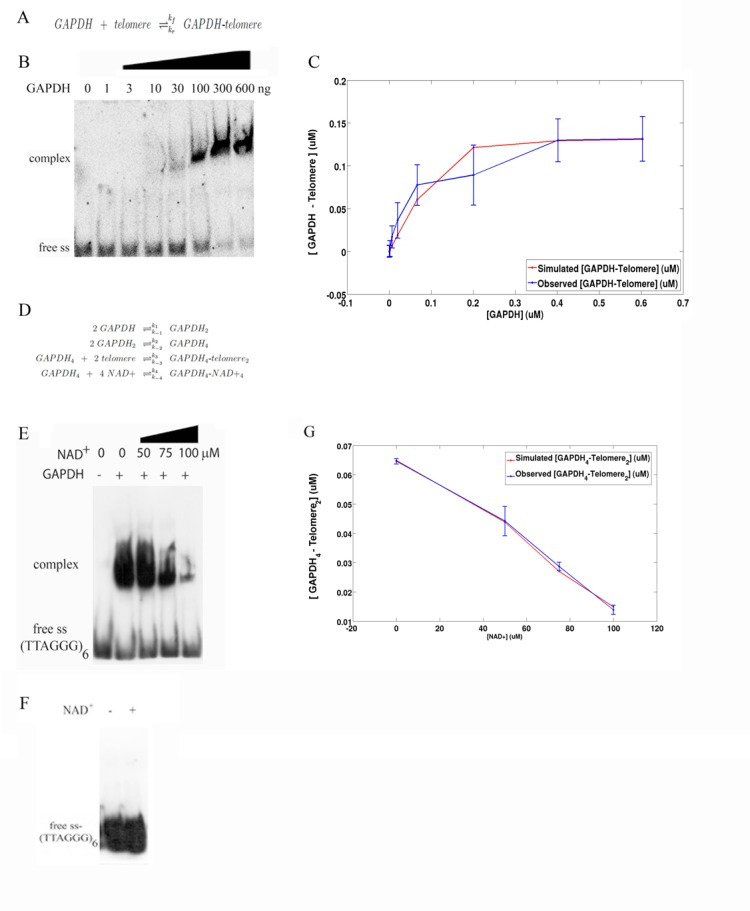
Mathematical model reproduces the association between GAPDH and telomere. (A) Chemical reaction that describes the dynamic equilibrium between the amounts of GAPDH, telomere and GAPDH-telomere complex. (B) 0.155μM of ss-(TTAGGG)6 labeled oligo were incubated with crescent amount of GAPDH. Samples were analyzed by EMSA. (C) Graph that describes the fraction of telomere bound to GAPDH as a function of the concentration levels of GAPDH. Each point of the blue line represents the quantification average of five independent biological experiments (presented on B). The red line is the result of a computational simulation that was obtained using a curve-fitting optimization on the system of differential equations presented in S1 Fig. (D) Chemical reactions that, coupled with the reaction depicted in A, describe the competition between NAD+ and telomeres for binding with GAPDH. (E) Labeled ss (TTAGGG)_6_ was incubated with 1.35 μM recombinant *T*. *cruzi* GAPDH in the presence of increasing concentrations of NAD^+^. The samples were analyzed by EMSA. (F) Labeled ss (TTAGGG)_6_ oligos were incubated in the presence (+) or absence (-) of 50 μM NAD^+^. The samples were subsequently analyzed by EMSA. (G) Graph that describes the fraction of 2 telomeres bound to tetramer GAPDH as a function of the concentration levels of NAD+. Each point of the blue line represents the quantification average of five independent biological experiments presented in E. The red line is the result of a computational simulation that was obtained using a curve-fitting optimization on the system of differential equations presented in S2 Fig.

We assessed the fraction of ss oligonucleotide G-rich strand bound to the rTcGAPDH protein as a function of the concentration of monomeric rTcGAPDH, in order to study the dynamic equilibrium between the strand bound to the protein and the free form of these two chemical species (Fig. 2A). To this end, we quantified the five independent replicates of the experiment showed in Fig. 2B. For each concentration of rTcGAPDH, we took the quantification mean and deviation. The obtained means, in their turn, were interpolated into a curve (Fig. 2C, blue line). In the sequence, the kinetics of the chemical reaction of Fig. 2A was described through the system of differential equations depicted in S1 Fig. Due to its simplicity we can assume that we can retrieve the approximate kinetic behavior by simply using these equilibrium data. Finally, we performed curve-fitting optimization to estimate the rate constants of the system in S1 Fig (Fig. 2C, red line). The initial concentrations and rate constants that were produced through this procedure are presented in Table 1. The ratio between the estimated reverse and forward rate constants (respectively, k_r_ and k_f_) is the estimated dissociation constant for the binding of ss oligonucleotide G-rich strand with GAPDH. Since simulated and experimental curves are in accordance, we suggest that the interaction between telomeric/oligonucleotide and rTcGAPDH follows ligand-receptor kinetic. We also observed that the experimental curve is in accordance with a curve simulating interaction of tetramer GAPDH with 2 telomeres (see below).

**Table 1:** List of initial concentrations and rate constants obtained through curve-fitting optimization on the system of differential equations presented in S1 Fig.

Initial concentration	Values
[GAPDH]	0, 0.00067, 0.00201, 0.0067, 0.0201, 0.067, 0.201, 0.402, 0.603
[GAPDH-telomere]	0
[telomere]	0.133

Rate constant	Value
k_r_	0.01
k_f_	1.3

### NAD^+^ competes with telomeric/oligonucleotide for rTcGAPDH association in a cell-free sytem

We tested if rTcGAPDH, similarly to human GAPDH is able to interact with telomeric DNA via its NAD^+^ binding domain. This hypothesis is feasible to be tested first because both humans and trypanosomes share the same telomeric DNA sequence and also because GAPDH from humans and trypanosomes share 44% identity. Thus, rTcGAPDH was incubated with ss telomeric oligonucleotide (TTAGGG)_6_ in the presence of equal or increasing concentrations of NAD^+^, and samples were subjected to EMSA analysis. We found that NAD^+^ inhibited the rTcGAPDH-telomerric/oligo interaction (Fig. 2E) in a dose-dependent manner and that the inhibition of complex formation was not due to an interaction between NAD^+^ and the telomeric DNA (Fig. 2F), suggesting that trypanosome GAPDH binds telomeres using its NAD^+^ binding domain. We quantified the five independent replicates of the experiment showed in Fig. 2E. For each concentration of NAD^+^, we took the quantification mean and deviation. The obtained means, in their turn, were interpolated into a curve (Fig. 2G, blue line). In the sequence, we tried to fit this obtained curve in a simulated curve using the stoichiometry of rTcGAPDH-telomeric/oligo interaction presented in Fig. 2A, but we could not reproduce the dynamics of the model (data not shown). Since it has already proposed that tetrameric mammalian GAPDH binds two molecules of telomere [8], we decide to build a mechanistic model reproducing this. We repeated curve-fitting optimization incorporating the dimerization and tetramerization GAPDH reactions, using the stoichiometry presented in Fig. 2D. The kinetics of the oligomerization reactions of Fig. 2D was described through the system of differential equations depicted in S2 Fig. Finally, we performed curve-fitting optimization to estimate the rate constants of the system in S2 Fig (Fig. 2G, red line), using as a priori information for the optimization procedure the rate constants estimated in the previous section. The initial concentrations and rate constants that were produced through this procedure are presented in Table 2. Since simulated and experimental curves are in accordance, we suggest that the interaction between telomere and GAPDH in *T*. *cruzi* follows the same model proposed for mammalian cells, where a tetrameric GAPDH associates with two telomeres. We also performed a negative control trying to fit the experiment with tetramer GAPDH with just one telomere and the curve did not match as well as tetramer GAPDH to two telomeres (S3 Fig).

**Table 2:** List of initial concentrations and rate constants obtained through curve-fitting optimization on the system of differential equations presented in S2 Fig.

Initial concentration	Values
[GAPDH]	1.35
[GAPDH_2_]	0
[GAPDH_4_]	0
[GAPDH-NAD+]	0
[GAPDH2-NAD+_2_]	0
[GAPDH4-NAD+_4_]	0
[GAPDH4-telomere_2_]	0
[NAD+]	0, 50, 75, 100
[telomere]	0.133

Rate constant	Value
k_-1_	1.43
k_1_	3.67e+04
k_-2_	27.7
k_2_	4.26e+04
k_-3_	1.99
k_3_	4.62e+04
k_-4_	1.86
k_4_	0.000104

### *T*. *cruzi* GAPDH associates with telomeric DNA *in vivo*

**Fig 3:**
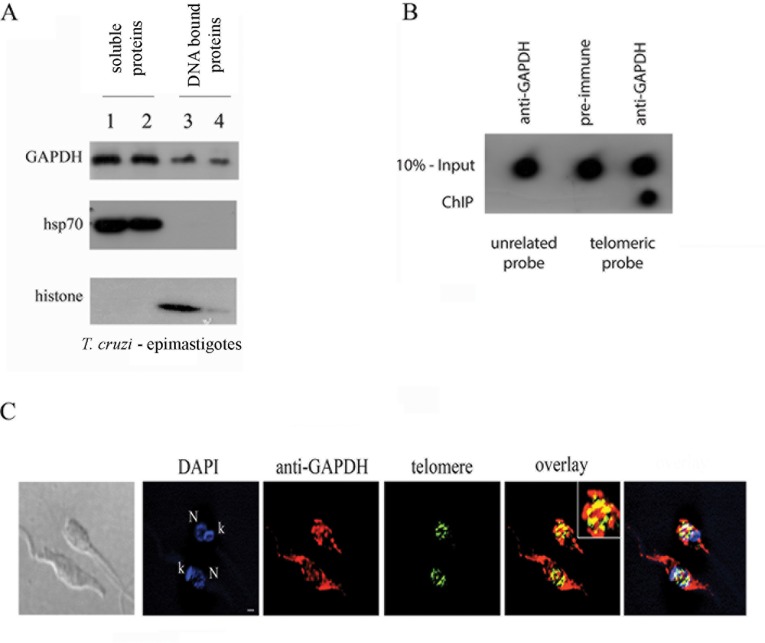
GAPDH associates with telomeric DNA *in vivo* in *T*. *cruzi* epimastigote cells. (A) Pellet from exponentially growing *T*. *cruzi* epimastigote cells was extracted using detergent-containing buffer (lane 1). The pellet was extracted again using the same detergent-containing buffer (lane 2). The pellet was digested with DNAse, the sample was centrifuged again, and the supernatant was saved and labeled as the DNA-bound protein fraction 1 (lane 3). The pellet was digested with DNAse again, the sample was centrifuged and the supernatant was saved and labeled as the DNA-bound protein fraction 2 (lane 4). The samples were analyzed by SDS-PAGE followed by Western blotting with anti-GAPDH, anti-hsp70 and anti-histone H4 antibodies. (B) *T*. *cruzi* epimastigote cells were submitted to a chromatin immunoprecipitation assay using an anti-GAPDH serum or pre-immune serum as a control. After immunoprecipitation, cross-links were reversed, and DNA was extracted and immobilized onto a nylon membrane. The membranes were hybridized to telomeric probes (CCCATT)_6_ or to nonspecific sequence probes (see material and methods). (C) FISH/IF assay where epimastigote cells were hybridized with telomeric probe (5’TTAGGGTTA3) (green) and incubated with anti-GAPDH antibody (red). N-nucleus, k-kinetoplast and bar is 1μm.

We further investigated whether *T*. *cruzi* GAPDH interacted with DNA *in vivo*. First, we performed a differential extraction previously established in our lab [22] to discriminate between soluble proteins and proteins bound to DNA by treating cells twice with detergent in order to get the soluble fraction. Then, the non-soluble fraction was digested with DNase I twice in order to get the DNA-bound proteins. As shown in Fig. 3A, GAPDH was present in the soluble fraction as expected and also in the DNA bound proteins fraction. As controls, heat shock protein (hsp70) and histone were used to discriminate between soluble and DNA bound proteins fractions. Because GAPDH was bound to DNA, we then tested whether it was associated with telomeric DNA using a chromatin immunoprecipitation assay. As shown in Fig. 3B, GAPDH bound *in vivo* the telomeric sequence in *T*. *cruzi* epimastigote cells. Pre-immune serum was used as a control and no telomeric DNA was detected in this sample. The amount of telomeric DNA detected after chromatin immunoprecipitation was 50% of the amount present in 10% of the input fraction, which represents 5% of the total telomeric DNA. Three independent experiments yielded closely estimated figures. To confirm, by another methodology, that GAPDH associates with telomeric DNA we conducted a FISH/IF assay, where replicative epimastigote cells were hybridized with telomeric probe and also labeled with anti-GAPDH antibody. The co-localization of these two signals (Fig. 3C) corroborated that GAPDH associates with telomere in *T*. *cruzi in vivo*.

### NAD^+^/NADH balance co-relates with GAPDH-telomere association

**Fig 4:**
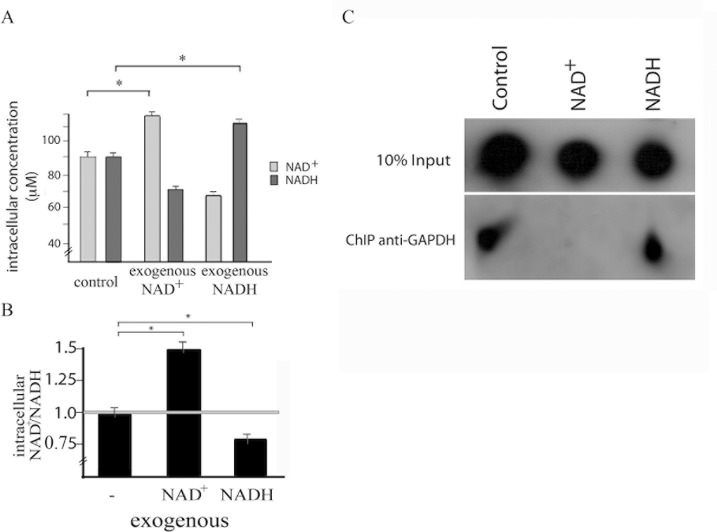
Exogenous NAD^+^ interrupts GAPDH-telomeric association in epimastigote cells. (A) and (B) *T*. *cruzi* epimastigote cells were maintained in the presence of NAD^+^ or NADH for 10 min. Afterwards, cells were lysed and the amount of intracellular NAD^+^ and NADH was quantified. Graph shows mean and standard deviation of five independent experiments. Statistic analysis was performed using 2way ANOVA, * means p < 0.001. (C) The same samples were subjected to a chromatin immunoprecipitation assay using an anti-GAPDH antibody. After immunoprecipitation, cross-links were reversed, and DNA was extracted and immobilized onto a nylon membrane. The membranes were hybridized to a telomeric probe (CCCATT)_6._

GAPDH is a NAD^+^ binding protein. The NAD^+^/NADH balance can control NAD^+^ binding proteins activity. We then investigated if the NAD^+^/NADH balance could control GAPDH-telomeric association. Therefore, epimastigote cells were maintained in the presence of NAD^+^ or NADH. The intracellular amount of NAD^+^ and NADH were quantified in order to show that adding NAD^+^ or NADH in epimastigote culture also changes their intracellular amount (Fig. 4A and 4B). Treated cells were submitted to chromatin immunoprecipitation using anti-GAPDH antibody and hybridizing the DNA obtained with telomeric probe. While we found telomere in control cells or cells treated with NADH, we could not find telomeric DNA in cells treated with NAD^+^ (Fig. 4C). These results have shown that exogenous NAD^+^ reduces GAPDH-telomere association.

**Fig 5:**
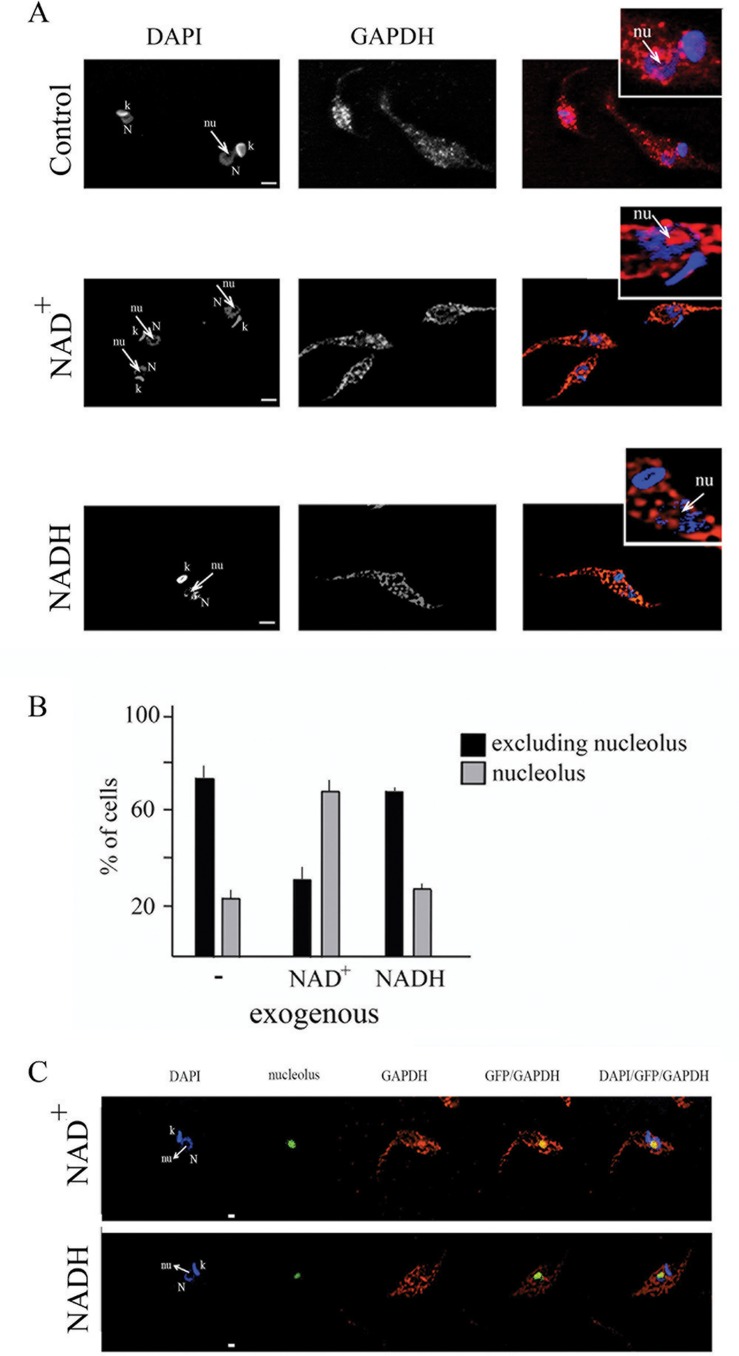
Exogenous NAD^+^ triggers translocation of GAPDH to nucleolus. (A) *T*. *cruzi* epimastigote cells were maintained in the presence of NAD^+^ or NADH for 10 min, fixed, permeabilized and incubated with anti-GAPDH antibody (red). Cells were also stained with DAPI (blue). N- nucleus, k-kinetoplast and nu- nucleolus. Bars are 2μm. (B) Percentage of cells presenting GAPDH constrained at nucleolar space or dispersed through the nuclear space was quantified. Graph shows media and stand deviation of three independent experiments (n = 50 in each experiment). (C) *T*. *cruzi* epimastigote cells expressing GFP (green) that localizes in the nucleolus were maintained in the presence of NAD^+^ or NADH for 10 min, fixed, permeabilized and incubated with anti-GAPDH antibody (red). Cells were also stained with DAPI (blue). N- nucleus, k-kinetoplast and nu- nucleolus. Bars are 1μm.

In order to see what happens with GAPDH in the nuclear space of NAD^+^ treated cells, we performed immunofluorescence with anti-GAPDH in control cells or cells maintained in the presence of exogenous NAD^+^ or NADH. We found that when cells were maintained in the presence of exogenous NAD^+^ GAPDH was translocated to nucleolar space (Fig. 5A and B). To confirm this result we used a parasite expressing green fluorescent protein (GFP) fused with the first 33 amino acids of the *T*. *cruzi* histone H2B. This hybrid protein is found exclusively as a large dot in the nucleolus of epimastigotes [23]. It is clear that GAPDH co-localized with GFP in NAD^+^ treated cells, while it did not not co-localized in NADH treated cells (Fig. 5C).

**Fig 6:**
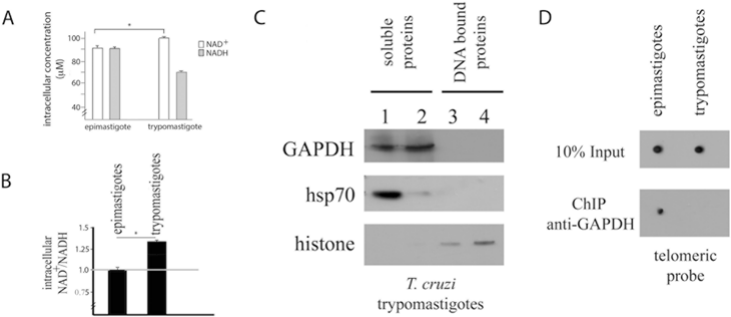
T. cruzi trypomastigote forms do not exhibit GAPDH-telomere association. (A) and (B) *T*. *cruzi* epimastigotes and trypomastigotes were lysed and the amount of intracellular NAD^+^ and NADH was quantified. Graphs show mean and standard deviation of five independent experiments. Statistic analysis was performed using 2way ANOVA, * means p < 0.001. (C) Pellet from trypomastigote cells was extracted using detergent-containing buffer (lane 1). The pellet was extracted again using the same detergent-containing buffer (lane 2). The pellet was digested with DNAse, the sample was centrifuged again, and the supernatant was saved and labeled as the DNA-bound protein fraction 1 (lane 3). The pellet was digested with DNAse again, the sample was centrifuged and the supernatant was saved and labeled as the DNA-bound protein fraction 2 (lane 4). The samples were analyzed by SDS-PAGE followed by Western blotting with anti-GAPDH, anti-hsp70 and anti-histone H4 antibodies. (D) Same samples were subjected to a chromatin immunoprecipitation assay using an anti-GAPDH antibody. After immunoprecipitation, cross-links were reversed, and DNA was extracted and immobilized onto a nylon membrane. The membranes were hybridized to a telomeric probe (CCCATT)_6_.

*T*. *cruzi* exhibits a complex life cycle in which different stages are constantly subjected to different environmental conditions, particularly different energy sources; thus, these organisms must display flexible metabolic patterns [24,25]. In particular, a metabolic switch from a metabolism based on glucose consumption to one based on amino acids consumption (particularly proline) happens along the host-cells invasion when the parasites face the intracellular environment [14]. As these changes occur along the parasite life cycle, we then tested whether the NAD^+^/NADH balance is also altered during the *T*. *cruzi* life cycle by comparing the intracellular levels of NAD^+^ and NADH obtained for both the insect-living replicative epimastigote form and the mammalian-living non-replicative trypomastigote form. We found that while epimastigote forms exhibited similar concentrations of NAD^+^ and NADH, trypomastigote forms exhibited higher levels of NAD^+^ (Fig. 6A). Because our observations suggested that trypomastigote cells present NAD^+^ concentrations higher than those of NADH, which does not favor GAPDH-telomere interaction, we tested whether GAPDH from trypomastigote forms could bind DNA (Fig. 6B) and or telomeric DNA (Fig. 6C). The results suggested that GAPDH did not associate with DNA and/or telomeric DNA in trypomastigote cells, confirming our previous hypothesis that the balance of NAD^+^/NADH is related to GAPDH-telomere association during the developmental cycle of *T*. *cruzi*.

**Fig 7:**
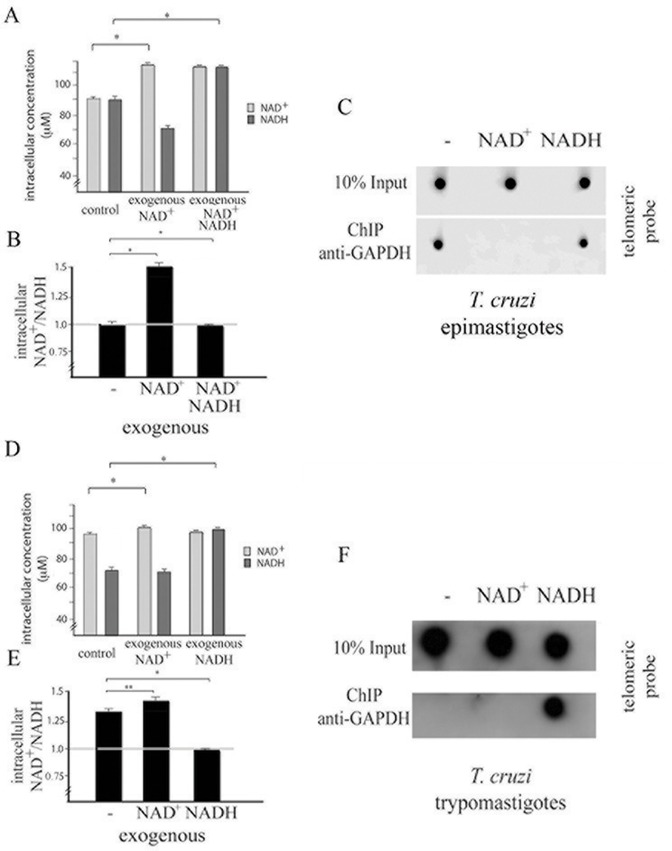
NAD^+^/NADH ratio is strongly related with GAPDH-telomere association in epimastigote and trypomastigote forms. (A) and (B) *T*. *cruzi* epimastigote cells were maintained in the presence of NAD^+^ for 10 min and then NADH was subsequent added (NAD^+^ NADH) for more 10 min. Afterwards, cells were lysed and the amount of intracellular NAD^+^ and NADH was quantified. Graph shows mean and standard deviation of three independent experiments. Statistic analysis was performed using 2way ANOVA, * means p < 0.001. (C) The same samples were subjected to a chromatin immunoprecipitation assay using an anti-GAPDH antibody. After immunoprecipitation, cross-links were reversed, and DNA was extracted and immobilized onto a nylon membrane. The membranes were hybridized to a telomeric probe (CCCATT)_6._. (D) and (E) *T*. *cruzi* trypomastigotes were maintained in the presence of NAD^+^ or NADH for 10 min. Afterwards, cells were lysed and the amount of intracellular NAD^+^ and NADH was quantified. Graph shows mean and standard deviation of three independent experiments. Statistic analysis was performed using 2way ANOVA, * means p < 0.001 and ** means p < 0.05. (F) The same samples were subjected to a chromatin immunoprecipitation as in (C).

To confirm that NAD+/NADH balance could be in fact involved with GAPDH-telemore association we performed two additional experiments. In the first one epimastigotes treated with NAD^+^ was subsequently treated with NADH and we could observe that the addition of NADH recovered the binding of GAPDH to telomeric region (Fig. 7A-C). In the second one, trypomastigote cells were treated with NAD^+^ or NADH and NADH brought about GAPDH onto telomeric DNA (Fig. 7D-F). Taken together, these results show that the lack of telomere binding we have observed in trypomastigotes might be attributed to the NAD^+^/NADH ratio.

## Discussion

Here, we show that *T*. *cruzi* GAPDH associates with telomeric DNA, which demonstrates that this very early divergent eukaryote produces multifunctional proteins and that GAPDH is a multitasking enzyme in this organism. It has already been demonstrated that the parasite has multifunctional proteins [26]. The evolution of multifunctional proteins may represent a mechanism by which cells expand their genomic repertoire. For an organism that few examples of alternative splicing have been reported [27,28], this course of action could be an effective cellular resource to expand the use of information present in its DNA sequences. As previously shown, in mammalian cells, GAPDH-telomere interactions are involved in the maintenance of genomic integrity because GAPDH protects chromosomal ends from degradation [7,8]. In *T*. *cruzi*, GAPDH binds single-stranded DNA, and one repeat of the TTAGGG sequence is enough to establish the GAPDH-telomere interaction. Telomeric sequences are exactly the same in mammals and trypanosomes, and therefore trypanosome GAPDH might bind to telomeres through the NAD^+^ binding domain of tetrameric GAPDH as demonstrated for mammalian GAPDH [8] and suggested by our results. The mechanism by which telomeric structure is protected in *T*. *cruzi* is poorly understood. Until recently, only the gene encoding for a putative TTAGGG repeat-binding factor (TRF) protein, essential for telomere protection in *Trypanosoma brucei* [29] was found in the *T*. *cruzi* genome database. Consequently, a deeply investigation is needed to precisely determine the role of GAPDH in *T*. *cruzi* telomere protection. Although it has been established that GAPDH is involved in multiple cellular pathways [2,30], the mechanism by which cells regulate its subcellular localization is still under investigation. Also, the mechanism involved in GAPDH cytosolic to nuclear translocation in trypanosomes remains unclear.

Throughout *T*. *cruzi* life cycle, this parasite is exposed to different environmental, shifts in temperature and glucose source, and as a consequence, different life cycle stages support differences in metabolic pathway activation [25]. The NAD^+^/NADH ratio is an important component of the redox state of a cell, which is a measurement that reflects both the metabolic activities and the health of cells [31]. Accordingly, we have shown here that the NAD^+^/NADH ratio changes during the *T*. *cruzi* life cycle. Moreover, we have shown that GAPDH associates with telomeric DNA in epimastigotes but not in trypomastigotes by chromatin immunoprecipitation assay. Analysis of chromatin binding proteins by mass spectrometry also showed GAPDH present in epimastigote, but not in trypomastigote fractions (manuscript in preparation). Because these forms are distinguished by their ability to replicate, it is possible that the telomeric protection is necessary only during the replicative epimastigote stage. To determine whether the NAD^+^/NADH redox ratio controlled GAPDH-telomere interactions, we altered the NAD^+^/NADH balance by treating epimastigote cells with exogenous NAD^+^ or NADH. It has previously been demonstrated that addition of exogenous NAD^+^ increases the intracellular levels of NAD^+^, indicating that extracellular NAD^+^ can cross the intact plasma membrane [32]. Accordingly, we showed that the addition of exogenous NAD^+^ altered the internal balance of NAD^+^/NADH in *T*. *cruzi* cells. Previous reports have indicated that cytosolic and nuclear NAD^+^/NADH redox ratios are similar [33,34]. Therefore, we inferred that the NAD^+^/NADH redox ratio we observed while measuring intracellular [NAD^+^] and [NADH] was the nuclear redox state. We also observed that the NAD^+^/NADH ratio was able to control GAPDH-telomere interactions in epimastigote form of *T*. *cruzi*, suggesting that GAPDH can sense the redox state, which subsequently controls whether it associates with telomeric DNA. The involvement of NAD^+^ in a chromosomal end protection mechanism was also observed in yeast [35]. Our data also suggests that treatment with exogenous NAD^+^ trigger translocation of GAPDH to nucleolus. The molecular base of this mechanism might be further investigated, but it has been already showed that *T*. *cruzi* nucleolus is able to sequester key factors of RNA metabolism in response to transcriptional stress [36] and therefore GAPDH translocation to nucleolus could be a kind of response to redox status alterations.

In conclusion, we showed that throughout its life cycle, *T*. *cruzi* exhibits different redox states. These states might be involved with the association between GAPDH and telomeres, and this association is most likely involved in the protection of chromosomal ends. Because *T*. *cruzi* is constantly exposed to different environmental stressors, it is important to explore the involvement of redox status in the control of physiological functions in this organism, the effects of which are clearly distinct during different stages of growth.

## Supporting Information

S1 Fig System of differential equations that describes the kinetics of the chemical reactions presented in Fig. 2A. (TIF)

S2 Fig System of differential equations that describes the kinetics of the chemical reactions presented in Fig. 2D. (TIF)

S3 Fig Mathematical simulation of association between 1 telomere with tetramer GAPDH. Graph that describes the fraction of 1 telomere bound to tetramer GAPDH as a function of the concentration levels of NAD+. Each point of the blue line represents the quantification average of five independent biological experiments. The red line is the result of a computational simulation that was obtained using a curve-fitting optimization on the system of differential equations.
(TIF)
