# Supplementary figures and images for: Glyceraldehyde 3-Phosphate Dehydrogenase-Telomere Association Correlates with Redox Status in Trypanosoma cruzi
Source: PLoS One. 2015 Mar 16;10(3):e0120896. doi: 10.1371/journal.pone.0120896 (PMC4361584; doi:10.1371/journal.pone.0120896)

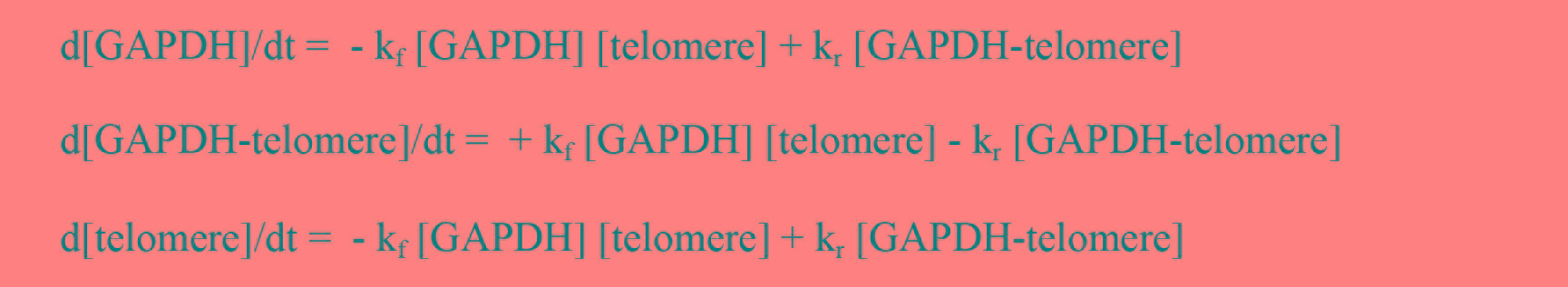

Supplement: S1 Fig — (TIF) [file pone.0120896.s001.tif]

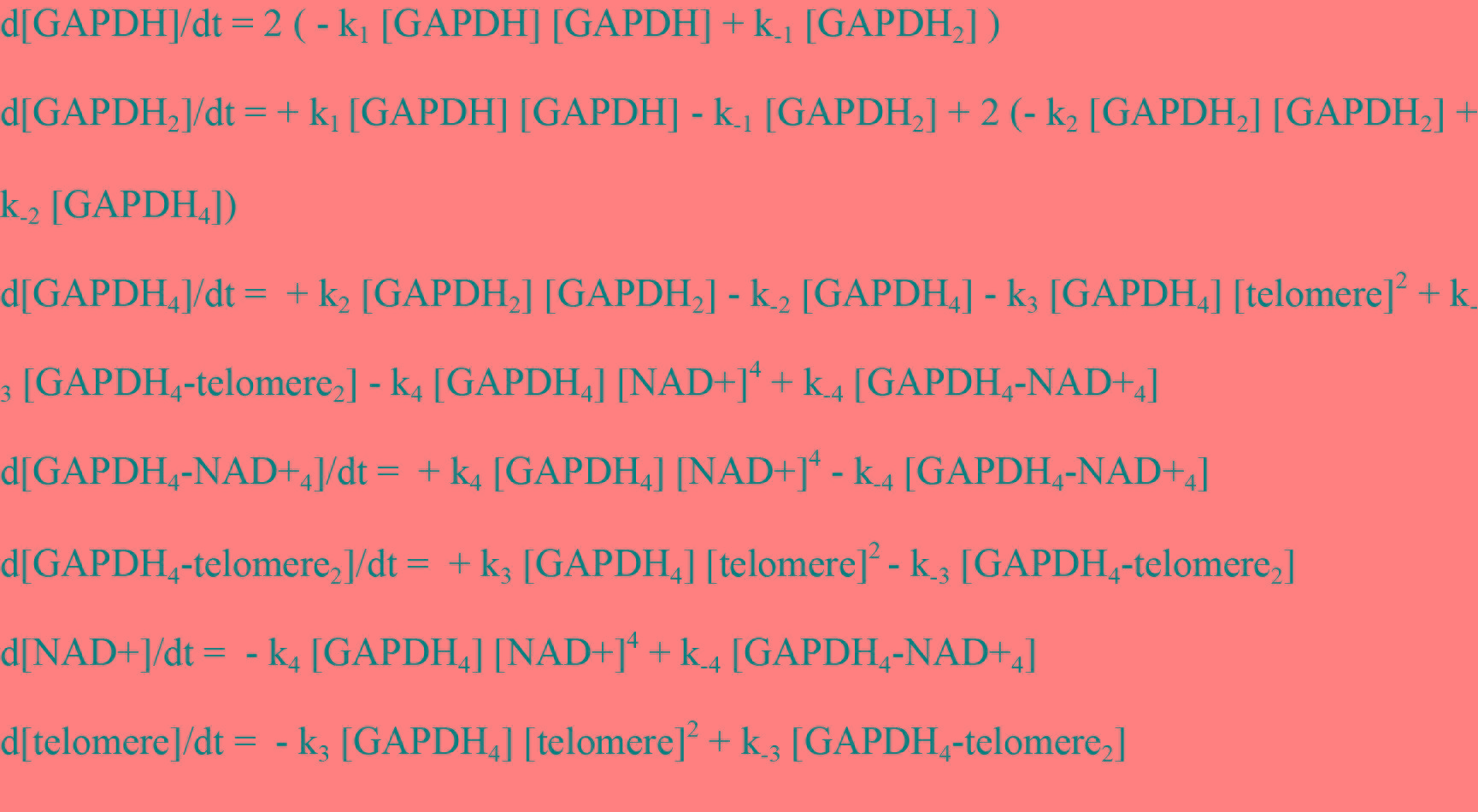

Supplement: S2 Fig — (TIF) [file pone.0120896.s002.tif]

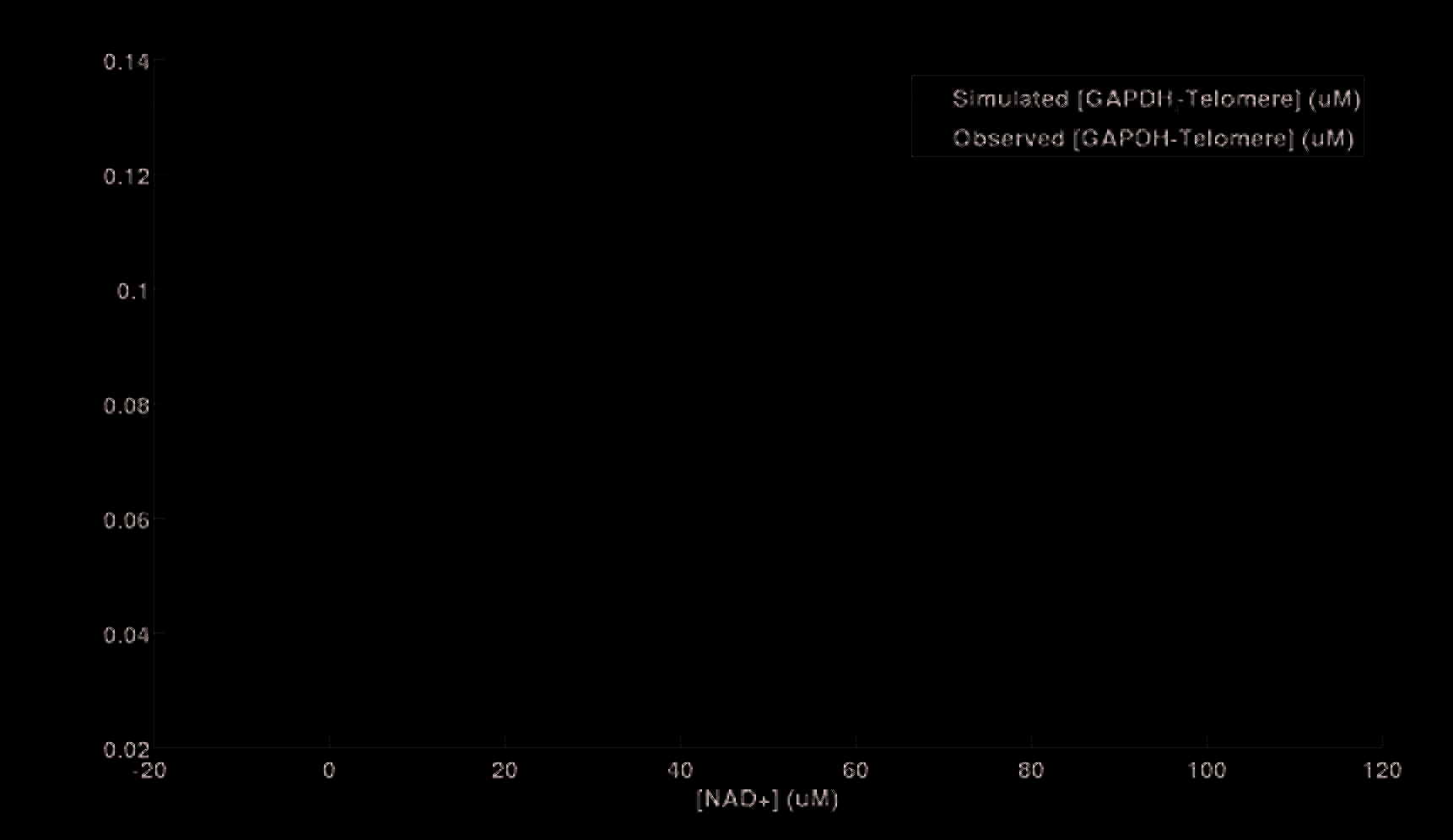

Supplement: S3 Fig — Graph that describes the fraction of 1 telomere bound to tetramer GAPDH as a function of the concentration levels of NAD+. Each point of the blue line represents the quantification average of five independent biological experiments. The red line is the result of a computational simulation that was obtained using a curve-fitting optimization on the system of differential equations. (TIF) [file pone.0120896.s003.tif]
